# Climate models fail to capture strengthening wintertime North Atlantic jet and impacts on Europe

**DOI:** 10.1126/sciadv.abn3112

**Published:** 2022-11-11

**Authors:** Russell Blackport, John C. Fyfe

**Affiliations:** Canadian Centre for Climate Modelling and Analysis, Environment and Climate Change Canada, Victoria, BC, Canada.

## Abstract

Projections of wintertime surface climate over Europe depend on reliable simulations of the North Atlantic atmospheric circulation from climate models. However, it is unclear whether these models capture the long-term observed trends in the North Atlantic circulation. Here, we show that over the period from 1951 to 2020, the wintertime North Atlantic jet has strengthened, while model trends are, on average, only very weakly positive. The observed strengthening is greater than in any one of the 303 simulations from 44 climate models considered in our study. This divergence between models and observations is now much more apparent because of a very strong jet observed over the past decade. The models similarly have difficulty capturing the observed precipitation trends over Europe. Our results suggest that projections of winter atmospheric circulation and associated precipitation over Europe may be unreliable because they fail to capture the response to human emissions or underestimate the magnitude of multidecadal-to-centennial time scale internal variability.

## Teaser

Models cannot capture the observed trends in winter North Atlantic circulation, calling into question future projections.

## INTRODUCTION

The atmospheric circulation in the North Atlantic is dominated by fluctuations in the jet stream, particularly in winter. Variations in the speed and latitude of the jet stream, or the North Atlantic Oscillation (NAO; which is known to reflect combined changes in jet speed and latitude), have a strong influence on surface European temperature and precipitation (*1*–*3*). Despite its importance for surface climate, how the North Atlantic circulation responds to increases in greenhouse gas concentrations is still highly uncertain (*4*, *5*).

Projections of how the atmospheric circulation may change in response to increased greenhouse gases are made using climate models. On average, climate models project that during winter, the North Atlantic jet stream will strengthen in the core and weaken along the northern and southern flanks (*6*–*8*). This has been referred as a “squeezing” or “narrowing” of the jet stream and has been shown to be driven by the opposing influences of the Arctic and the tropics (*7*). However, this response is weak and the responses in individual models differ considerably from the multimodel mean and from each other (*4*). Reducing the uncertainty in the circulation response would lead to more precise regional climate projections (*9*–*12*).

Evaluation of climate models relative to observations is essential to gain confidence in their projections. Comparing circulation trends in models and observations can be difficult because of the large role that internal variability plays on multidecadal time scales (*2*, *4*). On seasonal to decadal time scales, it has been argued that climate models underestimate predictable signals in the winter North Atlantic circulation, which could have implications for their responses to human emissions (*13*–*15*). Evaluation of climate models on even longer time scales similarly suggests potential issues. For example, from the 1960s to the 1990s, there was a tendency toward the positive phase of the winter NAO and a strengthening of the jet stream (*1*) that models struggled to capture (*16*–*18*). Subsequently, this tendency reversed toward a negative NAO and weaker jet stream coinciding with rapid sea ice loss and Arctic amplification (*19*–*21*). This led to questions about whether models can capture the large-scale circulation response to Arctic amplification and associated impacts on extremes (*22*–*24*). With that debate ongoing, the observed tendency toward a weaker jet and negative NAO has recently reversed once again (*25*, *26*). Questions surrounding the reliability of models, together with the recent shifts in short-term observed tendencies, motivate reexamining long-term trends in the North Atlantic atmospheric circulation and comparing them to the newest, state-of-the-art climate models.

In this study, we examine observed trends in winter North Atlantic circulation in reanalysis over 1951–2020 and compare them to trends in the suite of climate models from the Coupled Model Intercomparison Project Phase 6 (CMIP6), the latest phase of CMIP. The latest suite of models have reduced biases in the mean state of the winter North Atlantic circulation compared to previous phases of CMIP (*27*). Here, we go one very important step further than before and evaluate whether these latest models capture the observed long-term trends in North Atlantic jet speed, jet latitude, NAO, and the concomitant downstream impacts on precipitation change over Europe.

## RESULTS

### Spatial patterns of zonal wind and sea level pressure trends

**Fig. 1.**
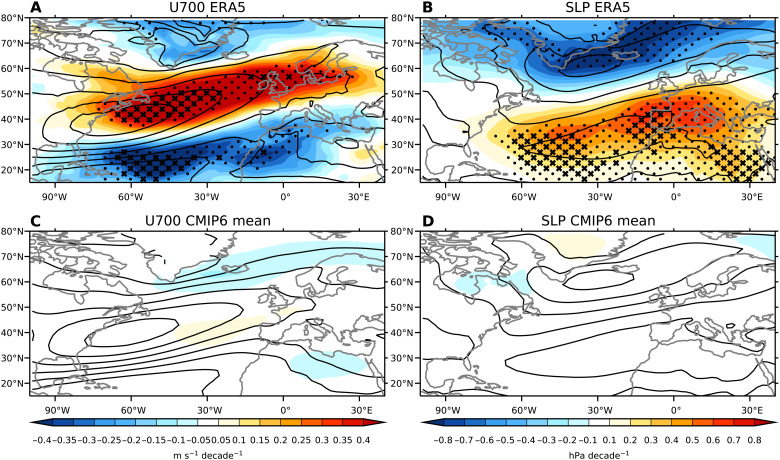
Observed and modeled trends in zonal wind and SLP. Spatial maps of winter trends over 1951–2020 for (**A**) U700 in ERA5, (**B**) SLP in ERA5, (**C**) U700 in the CMIP6 multimodel mean, and (**D**) SLP in the CMIP6 multimodel mean. Contours indicate the climatological values averaged over the 1951–2020 with contour intervals of 3 m/s for (A) and (C) and 5-hPa contour intervals for (B) and (D). Crosses in (A) and (B) represent regions where ERA5 trends are outside the CMIP6 model distribution, and dots represent regions where ERA5 trends are outside the 2.5 to 97.5% range from CMIP6.

We begin by comparing the spatial patterns of observed trends in atmospheric circulation from European Centre for Medium-Range Weather Forecasts Reanalysis version 5 (ERA5) to the multimodel mean from CMIP6 (see Materials and Methods). We focus on the trends in zonal wind at 700 hPa (U700) and sea level pressure (SLP). The trends are for winter averages (December-January-February) and calculated over the 1951–2020 period (the full time span of the ERA5 dataset). For U700 in ERA5 (Fig. 1A), there is a westerly trend over the region with the strongest climatological wind speeds, representing a strengthening in the core of the jet stream. There are also easterly trends along the southern flank of the jet stream and over the far north around Greenland. Similar results are found in trends from National Center for Environmental Prediction/National Center for Atmospheric Research (NCEP/NCAR) reanalysis (fig. S1A). The CMIP6 multimodel mean trends have some similar features compared to the observed trends (Fig. 1C). There is a westerly response over the jet stream, although the trend is strongest slightly equatorward and eastward compared to ERA5, and easterly trends over the northern flank and over North Africa. These features are similar to the response seen in the multimodel mean of future projections from CMIP5 and CMIP6 (*6*–*8*). However, the magnitude of the CMIP6 multimodel mean trends over the historical period from 1951 to 2020 is substantially weaker than observed.

The comparisons between observed and modeled trends in Fig. 1 are not like-for-like because the observed trends represent a single realization that includes internal variability, while the multimodel mean averages out internal variability. For this reason, we have also compared the observed trends to the simulated trends in each of the 303 presently available realizations from CMIP6. The cross-stippling in Fig. 1A represents where the observed trend falls outside the range of all CMIP6 model realizations, and the dotted stippling represents where the observed trends fall outside the 2.5 to 97.5% percentile range. The magnitudes of the observed westerly trends over the core of jet stream and easterly trends on the southern flank are outside the magnitude seen in any of the 303 realizations from CMIP6.

Trends in SLP, the variable upon which the NAO is based, show a similar picture to the trends in U700. In ERA5, there are decreases over the North Atlantic over the Icelandic low and increases over the southern Atlantic over the Azores high (Fig. 1B). Similar results are found in trends from NCEP/NCAR reanalysis (fig. S1B). This pattern projects strongly on a trend toward the positive phase of the NAO and is consistent with the observed trends toward strengthening of the jet stream. In the CMIP6 model mean, SLP trends are very weak, and there are few regions that show any notable trends over the North Atlantic (Fig. 1D). The observed trends over much of the regions of SLP increase and decrease are outside the 2.5 to 97.5% range of the CMIP6 models, with some of the positive trends entirely outside the model distribution.

### Time evolution of the North Atlantic circulation

**Fig. 2.**
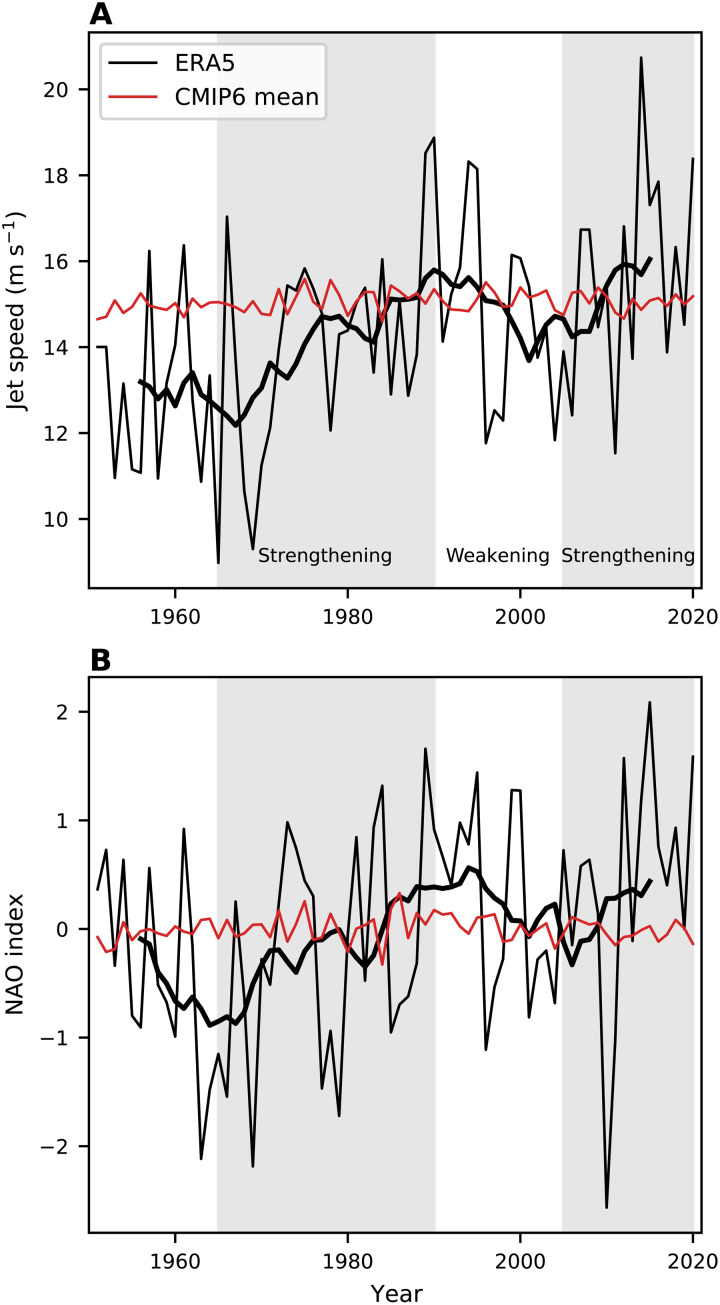
Time evolution of North Atlantic jet speed and NAO. Time series for (**A**) jet speed and (**B**) NAO index. The thin black line represents ERA5, the thick black line represents an 11-year running mean of ERA5, and the thin red line represents the CMIP6 multimodel mean. The gray shadings highlight the periods from 1965 to 1990 and from 2005 to 2020.

Motivated by previous work showing substantial multidecadal variability in the North Atlantic circulation (*28*, *29*), we next examine the time evolution of jet speed and NAO index (see Materials and Methods for full details) over the past 70 years (Fig. 2). The period between 1965 and 1990 (gray shaded on the left) shows a clear increase in the strength of the jet stream (Fig. 2A) and a trend toward a positive NAO (Fig. 2B) in ERA5. In the late 1990s and early 2000s, these short-term trends generated substantial discussion in the literature about whether these tendencies were externally forced or a result of internal variability and whether there was a large stratospheric involvement (*17*, *30*–*34*). Then, from 1990 to 2005, the jet stream weakened, and the NAO became more negative. This not only put into question the earlier debate about whether the jet strengthening and positive NAO tendencies from 1965 to 1990 represented a response to greenhouse gas forcing (and involvement of the stratosphere) but it also then launched a debate around whether this subsequent jet weakening was a response to greenhouse gas–induced sea ice loss and Arctic amplification (*19*–*21*), a debate that is still ongoing.

Bringing this discussion up to date using the latest observations, we note that the 1990–2005 tendency toward a weaker jet stream and negative NAO has reversed in recent years. The last decade was dominated by a stronger jet stream and a more positive NAO, with the 11-year period from 2010 to 2020 featuring the strongest jet of any 11-year period over the past 70 years. The relatively weak winter North Atlantic jet observed in the 2000s, which motivated the debate on the influence of the Arctic, was not particularly unusual in the longer context.

Against the considerable multidecadal variability shown in Fig. 2, there is a clear upward trend in the ERA5 reanalysis over the past 70 years in both jet speed and NAO. The observed trends over 1951–2020 are 0.52 m s^−1^ decade^−1^ (*P* = 0.0002) for jet speed and 0.15 decade^−1^ (*P* = 0.02) for the NAO index. In contrast, the CMIP6 multimodel mean shows very weak trends in both metrics. For jet speed, the models show a marginally statistically significant (*P* = 0.05) positive trend in multimodel mean jet speed (0.026 m s^−1^ decade^−1^), but this is about 20 times weaker than the observed trend from ERA5. The model trend in the NAO is very weak (0.003 decade^−1^) and not statistically significant, consistent with the lack of trends in SLP shown in Fig. 1. In contrast to jet speed and NAO, jet latitude shows little multidecadal variability and no statistically significant long-term trends in either the multimodel mean or in observations (fig. S2). It is clear that both the long-term trends and multidecadal variability in the NAO can be attributed to changes in jet speed and not jet latitude. These differences in multidecadal variability are consistent with previous work (*35*), showing that low-frequency variability in the NAO represents variability in jet speed.

For an even longer context, we have examined the time evolution of the jet speed in the ERA20C reanalysis, which extends back to 1900 (fig. S3). This is motivated by previous analysis of jet speed over the 20th century that showed a weaker jet speed that was relatively weak in the 1950s and 1960s (*28*, *29*). The longer time series shows that the early 20th century also featured a relatively strong jet, and that at the beginning of the period covered by ERA5, the jet was indeed relatively weak. However, the strength of the jet over recent decades is the strongest since at least the beginning of the 20th century. A caveat is that substantially fewer observations are used to constrain the reanalysis before 1950, and therefore, the uncertainty is likely larger than after 1950.

### Observed trends versus model distributions of trends

**Fig. 3.**
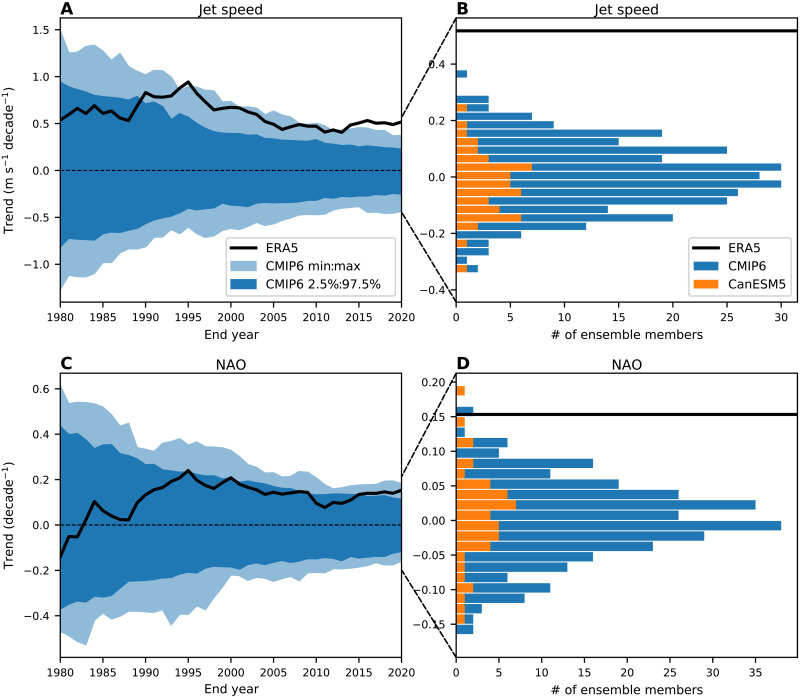
Comparison of observed circulation trends to model trend distributions. (**A**) The magnitude of linear trends in jet speed starting in 1951 as a function of end year. The black line represents trends in ERA5, the light blue shading represents the full range of trends from all CMIP6 model realizations, and the dark blue shading represents the 2.5 to 97.5% range. (**B**) Histogram of jet speed trends over 1951–2020 for all CMIP6 realizations (blue) and for the Canadian Earth System Model version 5 (CanESM5) (orange). The black line represents the 1951–2020 trends from ERA5. (**C**) As in (A) but for the NAO index. (**D**) As in (B) but for the NAO index.

We next examine how the observed trends compare to the distributions of model trends from individual CMIP6 realizations and how these observed trends and distributions of model trends have evolved over time (Fig. 3). For jet speed and NAO, the observed positive trends starting in 1951 and ending in the 1990s were on the extreme end or slightly outside the model distributions (Fig. 3, A and C). Since the mid-1990s, the magnitude of the observed trends has weakened but has remained positive and outside or near the 2.5 to 97.5 percentile ranges. Since the early 2010s, the magnitude of the observed trends in jet speed and NAO has increased and has become even more extreme relative to the model distribution than trends ending in the 1990s. For jet speed, the 1951–2020 observed trends are larger in magnitude than all 303 model simulations (Fig. 3B). Only 4 of 303 realizations have trends that are greater than half the magnitude of the observed trend. For the NAO, only 2 of 300 realizations show 1951–2020 trends of greater magnitude than the observed trend. These results show a clear discrepancy between model and observed trends in the North Atlantic even when the uncertainty due to model differences and internal variability are accounted for. Unlike the NAO and jet speed, the observed jet latitude trends have remained weak and inside the model distributions (fig. S4).

In addition to the CMIP6 distributions (which represents both model uncertainty and uncertainty due to internal variability), Fig. 3 (B and D) also shows the distributions from a 50-member ensemble of simulations from the Canadian Earth System Model version 5 (which only represents uncertainty due to internal variability). These distributions show a similar width compared to the full CMIP6 ensemble, suggesting that internal variability likely plays the dominant role in the CMIP6 spread in trends for the metrics examined here.

In fig. S5A, we compare the CMIP6 jet speed trend distributions for each overlapping 70-year trend to the observed trends from the time series of ERA5 concatenated with ERA20C. This shows that the 1951–2020 trend is the strongest magnitude trend and has the largest discrepancy with the model trends compared to any 70-year trend over the 1901–2020 period. In addition, varying the start year of the trend, while keeping the end year at 2020, reveals that all trends starting from the mid-1920s to the mid-1960s are outside the model distribution and that the 1901–2020 trend is on the extreme end of the model distribution (fig. S5B).

### Potential causes of the model-observation discrepancy

**Fig. 4.**
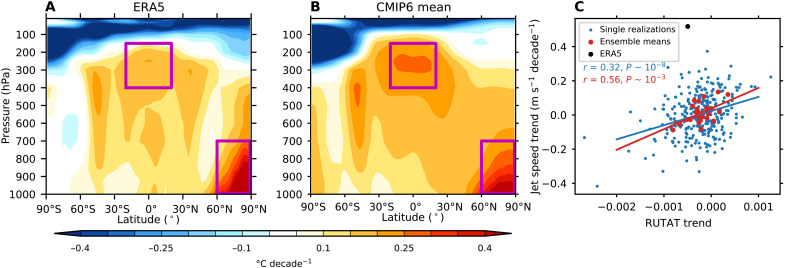
Links to tropical and Arctic temperature trends. (**A**) The zonal mean temperature trends (°C decade^−1^) averaged between 120°W and 60°E from ERA5 over 1951–2020. (**B**) As in (A), but for the CMIP6 multimodel mean. (**C**) Scatterplot of the trend in the ratio of upper troposphere tropical temperature to Arctic temperature (RUTAT) and jet speed trend. The black dot represents the ERA5 trends, the blue dots represent the individual ensemble members, and the red dots represent the ensemble means of all CMIP6 models with two or more ensemble members. Results from the linear regressions are indicated with the best line and correlations and *P* values. The purple boxes in (A) and (B) indicate the boxes used to calculate the RUTAT trend.

We next investigate whether differences in the tropical and/or Arctic warming between models and observations could explain the model-observation discrepancy in jet speed. Figure 4 (A and B) shows the zonal mean winter temperature trends over the hemisphere centered around the Atlantic Ocean (120°W to 60°E) for ERA5 and CMIP6 over 1951–2020, respectively. The temperature trends over the Arctic are similar between models and observations (0.31°C decade^–1^ in both), but the warming in tropical upper troposphere is weaker in ERA5 (0.16°C decade^–1^) than in the CMIP6 multimodel mean (0.24°C decade^–1^). This discrepancy in tropical upper troposphere temperature trends is consistent with previous analysis comparing trends from satellite observations and models (*36*–*38*). The differences in zonal mean temperature trends correspond to a stronger reduction in the north-south temperature gradient in observations compared to models. This may be expected to cause more of a weakening of the jet in observations compared to models; however, this is opposite to the clear strengthening that is seen.

To further investigate the link between the temperature trends and the circulation trends, Fig. 4C shows a scatterplot of the trend in jet speed and the trend in the ratio of tropical upper troposphere temperature to Arctic temperature (RUTAT). Both the individual ensemble members and the ensemble means for models with two or more members are plotted. Consistent with previous work investigating future projections (*7*), we find that, in historical trends, models with stronger tropical upper troposphere warming relative to Arctic warming tend to show more of a strengthening of the jet, with a moderate correlation (*r* = 0.56, *P* = 0.002) using the ensemble means. The trends from ERA5 show a weaker trend in the RUTAT than most models, which might suggest that we should see a weakening of the jet speed in observations, in contrast to the clear strengthening. Thus, instead of potentially explaining the model-observation discrepancy, analysis of the tropical and Arctic warming trends further highlights the discrepancy between observed and model trends in jet speed.

The projected response of the North Atlantic winter circulation has also been linked to changes in the strength of the winter stratospheric polar vortex (*6*, *39*, *40*) and North Atlantic sea surface temperature (SST) gradients (*7*, *41*, *42*). We find that there are links in the CMIP6 spread between the historical trends in jet speed and both the stratospheric polar vortex strength and the North Atlantic meridional SST gradient (fig. S6). However, the observed trends in these potential drivers are well within the CMIP6 spread (fig. S6) and thus cannot explain the observed trends in jet speed.

One potential explanation for the discrepancy is that the observed trend is a result of large changes in the observed Atlantic multidecadal variability (AMV) over the time period considered. Multidecadal variations in the jet speed have been linked to the AMV, and it has been shown that models may underestimate the atmospheric circulation response to the AMV (*43*). We confirm that the AMV is strongly correlated with the jet speed before ~2000 in observations (fig. S7). However, after ~2000, there is a clear divergence, with the AMV index predicting a weak jet speed, in contrast to the strong jet that has been observed. In addition, the trend in the AMV index is weak over the 1951–2020 period, strongly suggesting that the observed trend and the model-observation discrepancy is not linked to the AMV.

Another potential cause of the discrepancy is the biases in the climatological latitude and speed of the jet. We find that there is no link between the historical jet speed trend and climatological jet latitude or speed during winter (fig. S8). Furthermore, although the observed climatological jet latitude is more poleward than the CMIP6 model mean, both the jet speed and latitude are within the CMIP6 model spread. Thus, we conclude that biases in the jet speed and latitude are unlikely to be the cause of the discrepancy.

**Fig. 5.**
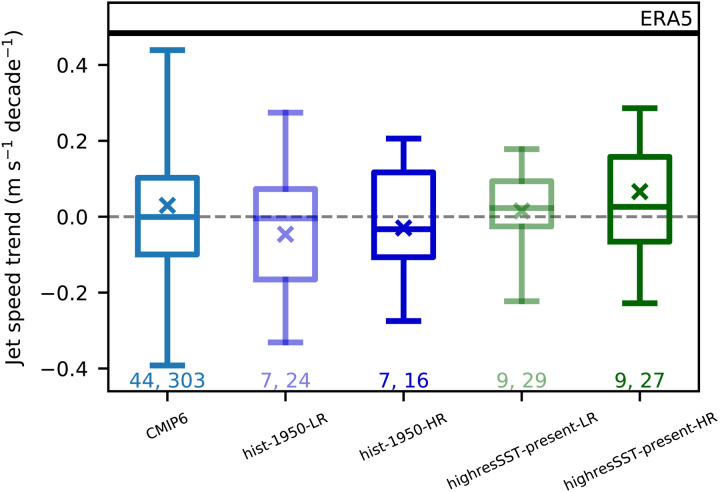
Jet speed trends in high-resolution models. Box-and-whisker plots for the jet speed trends over 1951–2014 from CMIP6, and both the low-resolution (LR) and high-resolution (HR) HighResMIP experiments. The box represents upper and lower quartile ranges, and the whiskers represent the minimum and maximum from all ensemble members. The lines in the boxes indicate the median from all ensembles, and the crosses represent the multimodel mean. The black line indicates the jet speed from ERA5 over 1951–2014. The two numbers at the bottom indicate the total number of models (left) and total number of ensemble members (right) from each experiment.

Last, we examine whether atmospheric model resolution could play a role in the discrepancy between models and observations using two experiments from the High Resolution Model Intercomparison Project (HighResMIP) project (*44*). The first, called “hist-1950,” is a coupled ocean-atmosphere experiment with historical forcing over 1950–2014. The second experiment, called “highresSST-present,” consists of an atmosphere model forced with the observed SST and sea ice over 1950–2014. To isolate the impact of model resolution, each model and experiment was done with both a high- and low-resolution version of the model. A complete list of the models, number of ensemble members, and resolutions is shown in table S2. Figure 5 shows the spread in the jet speed trends from CMIP6 and the high- and low-resolution versions for each experiment. We find little difference in the jet speed trends or in the spread of the trends between the high- and low-resolution versions of the models. We also find only a small difference in the trends when forced by observed SST, indicating that the biases in the SST are unlikely to explain the differences between models and observations.

### Impacts of changes in North Atlantic circulation on European precipitation

**Fig. 6.**
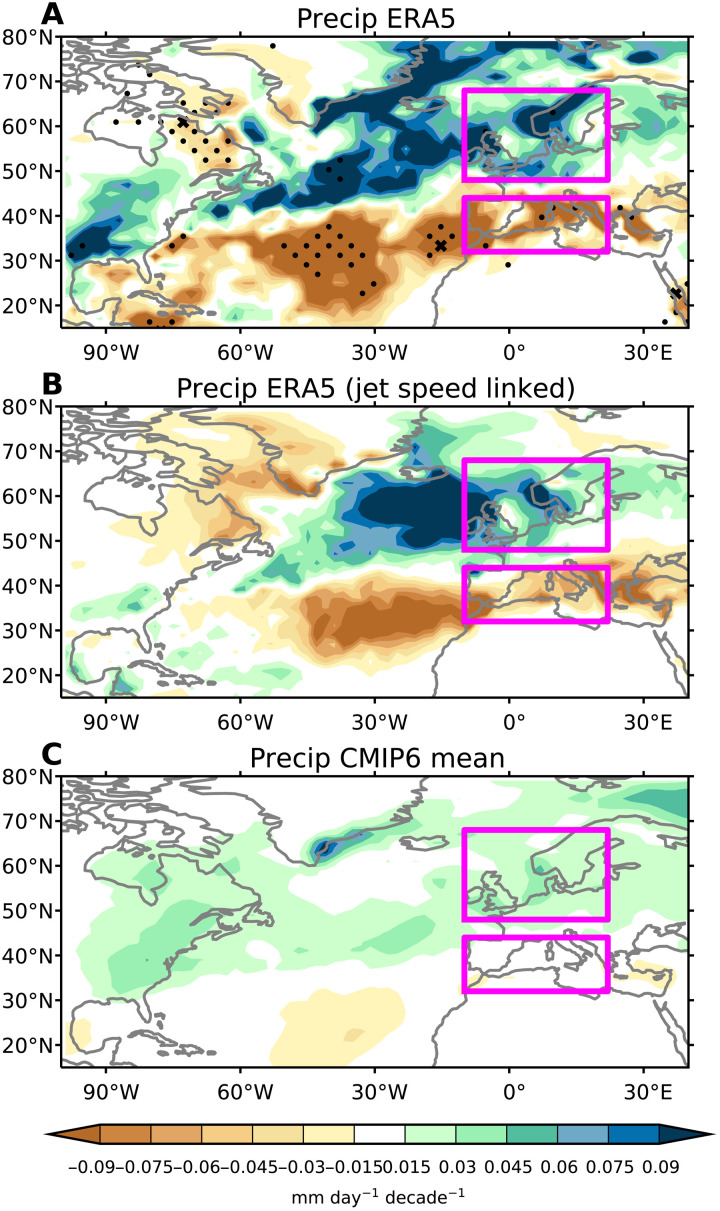
Precipitation trends and links to jet speed trends. (**A**) Spatial maps of winter precipitation trends from ERA5 over 1951–2020. Crosses represent regions where ERA5 trends are outside the full CMIP6 model distribution, and dots represent regions where ERA5 trends are outside the 2.5 to 97.5% range from CMIP6. The purple boxes indicate the regions used for Northern Europe and Southern Europe. (**B**) The precipitation trends from ERA5 that are linked with the jet speed trend (see Materials and Methods). (**C**) As in (A) but for the CMIP6 multimodel mean.

We next examine the impact the circulation trends documented above have had on precipitation over Europe, which is just one example of a potential societally relevant impact on surface climate. Associated with the trends toward a stronger jet stream and more positive NAO in ERA5, we find increases in precipitation over most of Northern Europe and decreases in precipitation over the Southern Europe and the Mediterranean region (Fig. 6A). Figure 6B shows the precipitation trends that are linked to the jet speed trend (see Materials and Methods). The pattern of precipitation trends linked to the jet speed trends is similar to the overall trends in Fig. 6A, with increases over Northern Europe and decreases over Southern Europe and the Mediterranean. About 60 to 70% of the total precipitation trends over each of these regions can be attributed to the jet speed trend. The time evolution of precipitation averaged over Northern Europe and Southern Europe show similar multidecadal variability to the atmospheric circulation (fig. S9 compared to Fig. 2). The CMIP6 mean also shows increases over Northern Europe and decreases over Southern Europe, but with much weaker magnitudes (Fig. 6C).

**Fig. 7.**
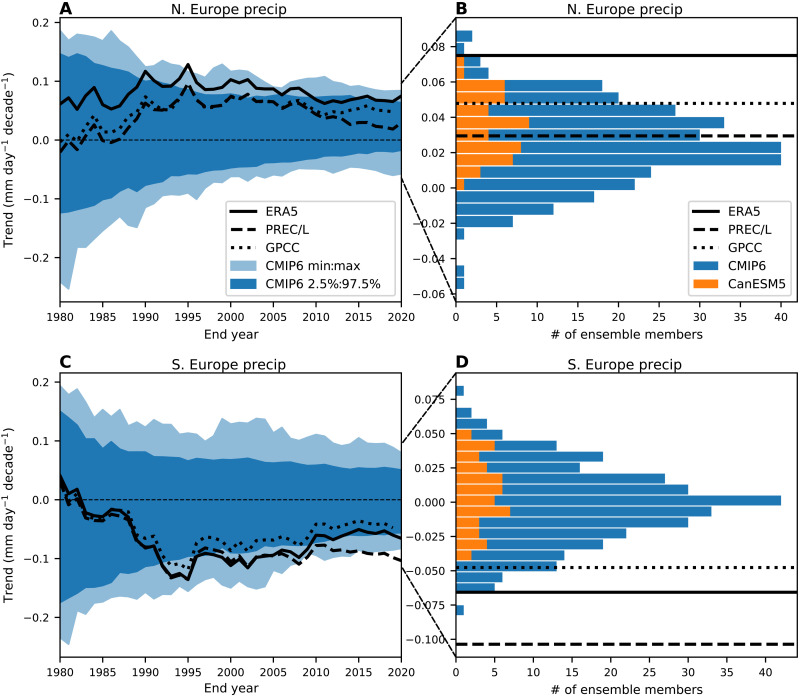
Comparison of observed precipitation trends to model trend distributions. (**A**) The magnitude of linear trends in Northern European precipitation (10°W to 22°E, 48°N to 68°N; land only) starting in 1951 as a function of end year. The solid black line represents trends in ERA5, the dashed black line represents observed trends from PREC/L, and the dotted black line represents observed trends from GPCC (only up to 2019). The light blue shading represents the full range of trends from all CMIP6 model realizations, and the dark blue shading represents the 2.5 to 97.5% range. (**B**) Histogram of Northern European precipitation trends over 1951–2020 for all CMIP6 realizations (blue) and for CanESM5 (orange). The black line represents the 1951–2020 trends from ERA5, the dashed black line represents the 1951–2020 trends from PREC/L, and the dotted black line represents the 1951–2019 trends from GPCC. (**C**) As in (A) but for Southern European precipitation (10°W to 22°E, 32°N to 44°N; land only). (**D**) As in (B) but for Southern European precipitation.

Figure 6A shows that there are only a few grid points where the ERA5 precipitation trends lie completely outside the distributions of model trends. Consistent with the circulation trends, the averages of ERA5 precipitation over Northern Europe and over Southern Europe lie at the extreme end of the model distributions (Fig. 7), although there are some disagreements with observational datasets, which are discussed in more detail below. We also note that precipitation decreases over northeastern Canada are linked with the circulation trends (Fig. 6B). These trends are opposite to what is predicted by the CMIP6 multimodel mean and are completely outside the model distribution (fig. S10). The time series of northeastern Canada precipitation also follows similar multidecadal variability to the circulation time series (compare fig. S11 with Fig. 2).

To verify the precipitation trends in ERA5, we have also examined gridded observations based on gauge data from the Precipitation Reconstruction over Land (PREC/L) and the Global Precipitation Climatology Centre (GPCC). The spatial patterns of the trends over land are similar (fig. S12), but the magnitudes show some differences with ERA5 and with each other (Fig. 6). These observations show weaker trends over Northern Europe that are in better agreement with the models. However, the Southern Europe precipitation trends from PREC/L are even farther outside the model distribution, and GPCC is still on the extreme end of the distribution.

## DISCUSSION

One potential explanation for the model-observation discrepancy in the North Atlantic circulation trends over the past 70 years is that the observed trends are a result of long-term unforced variability that is not captured by climate models. Previous work has shown that climate models underestimate the multidecadal variability of the North Atlantic climate (*18*, *43*, *45*–*49*), and this is particularly apparent in the multidecadal variability of the speed of the North Atlantic jet (*28*). We also find substantial multidecadal variability in the jet speed in observations; thus, the recent strengthening may be a manifestation of this low-frequency variability. However, the discrepancies found in previous analysis have been shown over trends of 30 years or less and are potentially linked with the AMV. The discrepancies that we find over 70-year trends are substantially longer than what has been found previously and cannot be explained by the AMV. It is possible that the discrepancy could be explained by internal variability on even longer time scales that models do not capture, but it is not clear what the source of this centennial time scale internal variability would be.

Another concerning interpretation of the discrepancies uncovered here is that the observed trends have a substantial contribution from anthropogenic forcing, and models are unable to capture this important response now and potentially in the future. We found a number of results that may suggest the possibility of a forced response in the observed trends. First, the spatial pattern of the observed trends in zonal winds do appear broadly similar to the squeezing of the jet that is seen in simulated trends in response to historical forcing and to projected forcing (*6*–*8*) in the multimodel mean. Furthermore, the 70-year trends in jet speed over the 1951–2020 period have the largest magnitude and show the largest discrepancy with models compared to any 70-year trend since at least the beginning of the 20th century. We also note that this discrepancy becomes more apparent when data from the most recent decade are included, data that were not included in earlier studies examining long-term variability of the jet (*28*, *29*). The fact that the strongest trends have coincided with the largest anthropogenic greenhouse forcing may suggest the possibility that this is a forced response. However, the fact that observed trends starting in the early 20th century are weaker and in better agreement with the models may suggest that the recent trends are not a result of external forcing. This, combined with the lack of physical understanding of the long-term trends and known deficiencies of the models in reproducing the observed multidecadal variability over the North Atlantic, makes it difficult to attribute the recent trends to anthropogenic forcing with any level of confidence.

Previous modeling studies have shown that the winter North Atlantic circulation response to future greenhouse gas increases depends on a “tug-of-war” between the tropics and the Arctic (*7*, *50*–*52*). Specifically, Arctic warming in the lower troposphere drives weakening of the jet stream, while tropical upper tropospheric warming drives a strengthening, leading to cancelations and weak responses overall. Some have argued that observational evidence suggests that the Arctic has been the dominant driver (*23*, *53*), in contrast to what is predicted by models, and that the jet stream may weaken in response to continued greenhouse gas emissions. However, these conclusions are based on relatively short periods between the late 1980s and early 2010s. Investigating the longer time series, by both extending the analysis back in time and updating with more recent data, presents a very different picture. The strengthening observed over the past 70 years, including the very strong jet stream in the recent decade, is not consistent with the Arctic being the dominant driver of changes in the North Atlantic circulation or with models underestimating the role of the Arctic. In contrast, the discrepancy that we show between models and observations would suggest that models may be underestimating the circulation response to tropical warming or overestimating the response to Arctic warming.

We found that the model-observation discrepancy in the circulation trends cannot be explained by discrepancies in trends in drivers such as tropical and Arctic warming, stratospheric polar vortex strength, or North Atlantic SST gradient. This would suggest that either another driver is responsible or the response to one or more of these drivers is misrepresented in the models. One potential explanation for the latter is that feedbacks between transient waves and mean circulation (so called eddy feedbacks) are too weak in models. This has been proposed as one possible reason for the underestimation of predictable signals in seasonal and decadal predictions (*13*–*15*, *54*). Weak eddy feedbacks could lead to an underestimation of the response to both low-frequency ocean variability and external forcing. Thus, it is possible that two interpretations are not mutually exclusive. That is, that the discrepancy in trends that we identified here could be due to a combination of the models underestimating both the low-frequency internal variability and the response to external forcing.

Some previous single-model studies have shown that increasing atmospheric resolution leads to a stronger circulation and associated precipitation response to projected forcing (*55*, *56*). However, using a multimodel ensemble of high-resolution models, we find little impact of increasing model resolution on jet speed trend over the historical period. The small impact of increasing resolution is consistent with recent studies that find little impact of increasing resolution in response to North Atlantic SST variability (*57*) and sea ice loss (*58*). Although we find limited impact of increasing resolution, it is possible that even higher resolutions (<25-km horizontal resolution) are needed to see larger differences.

Our results have important implications for climate projections over the North Atlantic, including Europe. If the observed jet strengthening has a strong contribution from greenhouse gas emissions and models do not capture this, then the model projections of the circulation and associated impacts are likely to be unreliable. For example, if the strengthening of jet stream continues, then winter precipitation is likely to continue to increase over Northern Europe and decrease over Southern Europe much faster than projected by the CMIP6 models. Even if the discrepancy in trends is because of discrepancies in low-frequency internal variability, rather than a response to external forcing, this would still imply that model projections would be unreliable. This is because models will underestimate the chance of internally driven long-term trends in circulation and associated impacts. Understanding the cause(s) of this discrepancy will be crucial to obtaining reliable projections.

In summary, we have compared observed and simulated trends of the wintertime North Atlantic atmospheric circulation over the 1951–2020 period. In reanalysis, despite substantial multidecadal variability, there is a clear trend toward strengthening of the jet stream and toward the positive phase of the NAO. In contrast, the multimodel mean trends in both the strength of the jet stream and the NAO are very weak to nonexistent. The magnitude of the observed trend in the jet stream speed is outside of the distribution of the model trends from 303 realizations from 44 models from CMIP6. Building on work showing that models underestimate predictable signals on seasonal to decadal time scales (*13*), this discrepancy shows that models are also not able to capture observed trends on the 70-year time scale. It is not clear whether this is indicative of issues with how the models respond to external forcing, the inability of the models to capture low-frequency internal variability, or a combination of both. Whatever the cause or causes, our results show that regional climate projections of the winter atmospheric circulation and associated impacts over Europe that rely on CMIP6 models may not be reliable.

## MATERIALS AND METHODS

### Reanalysis and models

For observed trends, we examine ERA5 reanalysis (*59*) over the 1950–2020 period. We use monthly averaged, zonal wind speed at 700 hPa (U700), SLP, precipitation, temperature, and SST data over the winter season defined as the December-January-February average. The labeled year refers to year for January and February (e.g., 2020 refers to average over December 2019, January 2020, and February 2020). To confirm the robustness of our results, we compared circulation trends in ERA5 and NCEP/NCAR reanalysis (*60*). We also examine the jet speed using the ERA20C reanalysis (*61*), which covers the period from 1900 to 2010. ERA20C reanalysis only assimilates surface pressure and marine wind observations. The mean jet speeds are bias-corrected against the ERA5 reanalysis using the common time period (1951–2010). A concatenated time series was created with the bias-corrected ERA20C jet speed from 1901 to 1950 and ERA5 from 1951 to 2020.

The precipitation data that we use are based on gauge data from PREC/L (*62*) and GPCC v2020 (*63*). The GPCC data are only available up to 2019. The SST data are from HadISST1.1 (*64*).

We compare the reanalysis results to the models from the CMIP6 archive. These consist of 303 realizations from 44 models (see table S1 for a complete list). Note that the exact number of realizations and models differs slightly for the different variables. These simulations are forced with historical external forcings from 1850 to 2014 and then projected forcings using the SSP2-4.5 scenario from 2015 to 2100, but we only examine the period from 1900 to 2020. We also use data from the HighResMIP (*44*) over the 1951–2014 period from the hist-1950 and highresSST-present (see table S2 for a complete list of models used). For models that have more than one ensemble member, the only difference between realizations is internal variability. Multimodel means are determined by first calculating the ensemble mean for each model and then averaging over all models. Similar results are found if instead only one ensemble member from each model is used. All data are interpolated to a common 2° (latitude) × 2° (longitude) grid before performing any analyses.

### Metrics and analysis

The speed and latitude of the North Atlantic jet stream are calculated similar to a previous analysis (*50*), with some minor differences. At each longitude between 60°W and 0°, we fit a parabola around the seasonal averaged, maximum U700 speeds found between 15°N and 75°N. The parabola is fit around the two points on either side of the maximum (five points in total). The maximum of the resulting function is the jet speed, and the latitude of this maximum is the jet latitude. If the maximum speed occurs at the northern or southern edge, then the speed and latitude are ignored for that longitude. The zonal mean of these quantities are then taken. Calculating the speed and latitude at each longitude before calculating the zonal mean removes the possibility of artifacts due to the tilt of the jet stream with latitude. However, we find similar results if we first calculate the zonal mean of the U700 fields before calculating the jet speed and latitude.

We calculate the NAO index similar to previous analysis (*14*). First, the seasonal mean SLP is averaged in two boxes, one around Azores (28°W to 20 W, 36°N to 40°N) and the other around Iceland (25°W to 16°W, 63°N to 70°N). These averages are normalized by their SD and differenced before removing the time mean and normalizing by the SD again. The precipitation time series and trends are examined for averages (land only) over Northern Europe (10°W to 22°E, 48°N to 68°N), Southern Europe (10°W to 22°E, 32°N to 44°N), and northeastern Canada (75°W to 60°W, 50°N to 62°N). The upper tropospheric temperature is defined as the average temperature between 20°S to 20°N and 400 to 150 hPa. The Arctic temperature is defined as the average over 60°N to 90°N and 1000 to 700 hPa. The AMV index is defined as the difference between SSTs averaged over the North Atlantic (0° to 60°N, 0° to 80°W) and the global mean SST (60°S to 60°N) (*65*).

To calculate the precipitation trend that is linked with the jet speed trend (Fig. 6B), we first remove the linear trend from the precipitation field and jet speed time series. We then regress the precipitation time series at each grid point against the jet speed time series. This represents the precipitation anomalies linked with changes in jet speed in interannual variability. This field is then multiplied by the magnitude of the jet speed trend.

Statistical significance is assessed using the two-sided Student’s *t* test. To account for autocorrelation in the time series data, the degrees of freedom are reduced using the effective sample size (*66*)

$$N_{\text{eff}}=N\frac{1-r}{1+r}$$

where *N*_eff_ is the effective sample size, *N* is the sample size, and *r* is the lag 1 autocorrelation.
