# Supplementary material for: Climate models fail to capture strengthening wintertime North Atlantic jet and impacts on Europe
Source: Sci Adv. 2022 Nov 11;8(45):eabn3112. doi: 10.1126/sciadv.abn3112 (PMC9651855; doi:10.1126/sciadv.abn3112)
Supplement: Supplementary file 1 — Figs. S1 to S12 Tables S1 and S2 [file sciadv.abn3112_sm.pdf]

Science Advances
AAAS

Supplementary Materials for

Climate models fail to capture strengthening wintertime North Atlantic jet and impacts on Europe

Russell Blackport and John C. Fyfe

Corresponding author: Russell Blackport, russell.blackport@ec.gc.ca

Sci. Adv. **8**, eabn3112 (2022)
DOI: 10.1126/sciadv.abn3112

This PDF file includes:

Figs. S1 to S12
Tables S1 and S2

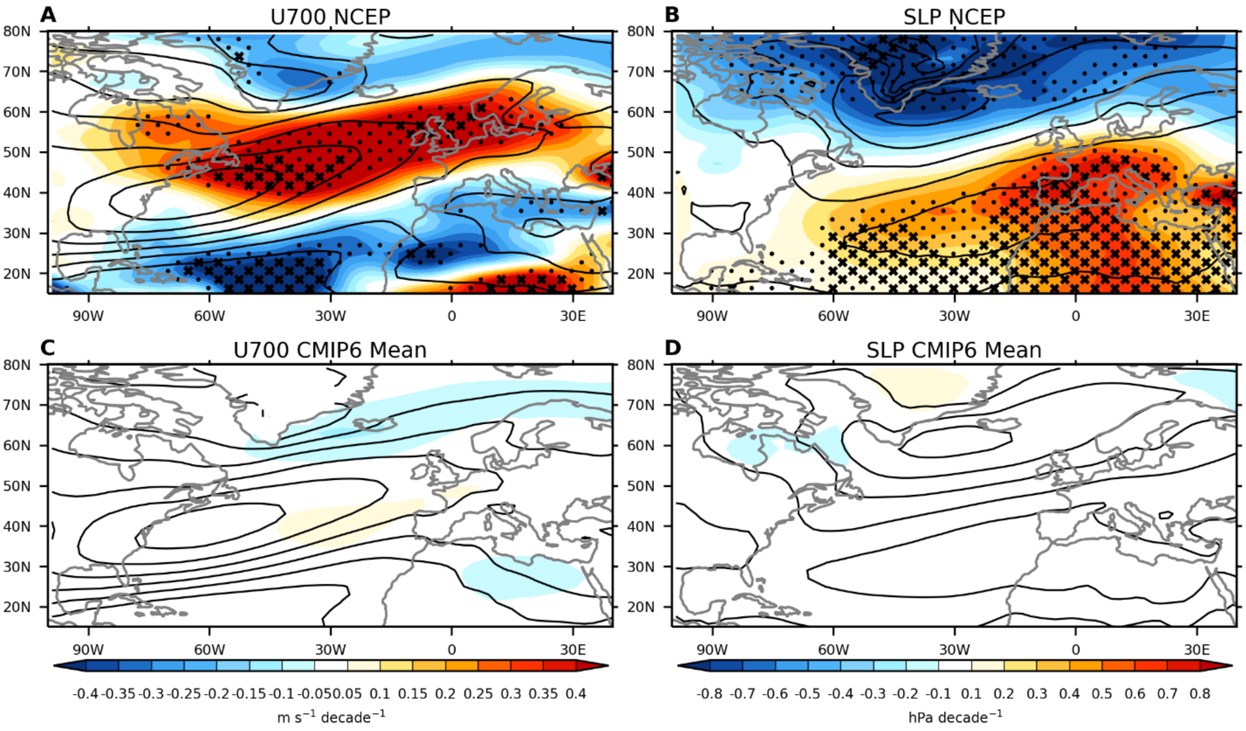

Fig. S1. Observed and modelled trends in zonal wind and SLP. As in Figure 1, but using NCEP/NCAR reanalysis.

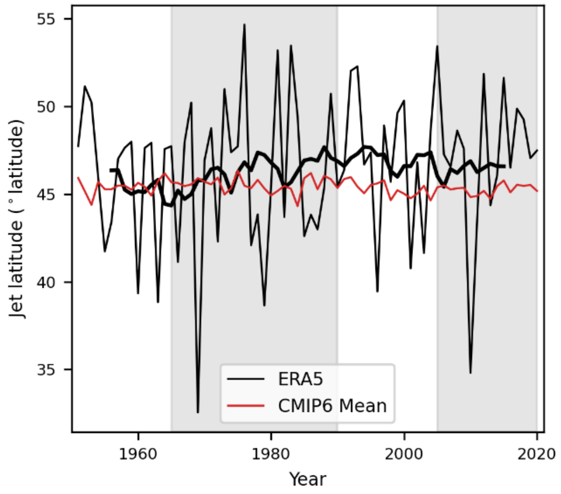

Fig. S2. Time evolution of North Atlantic jet latitude. As in Figure 2A, but for jet latitude.

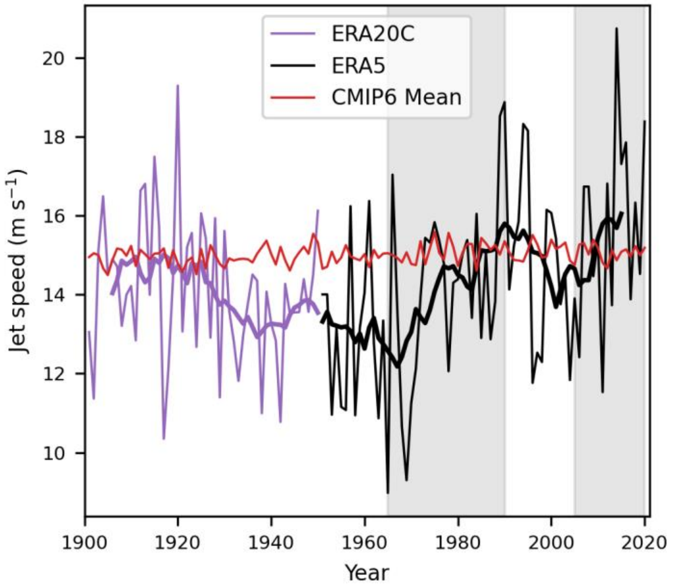

Fig. S3. **Time evolution of North Atlantic jet speed from ERA20C**. As in Figure 2A, but the jet speed time series from ERA20C reanalysis is included for the 1901-1950 period in purple.

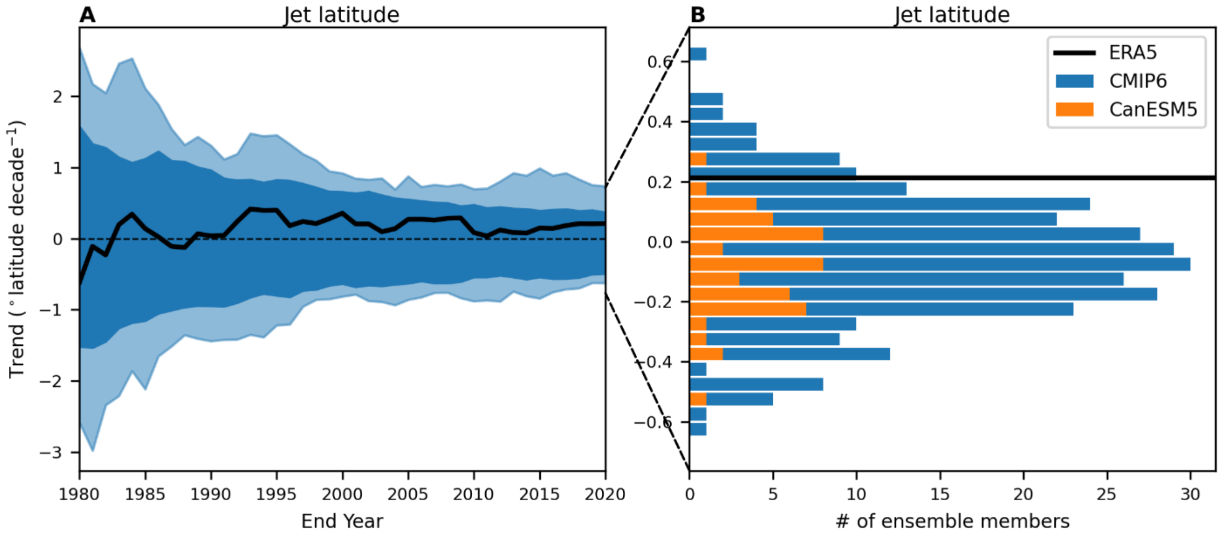

Fig. S4. Comparison of observed jet latitude trends to model trend distributions. As in Figure 3A, but for jet latitude.

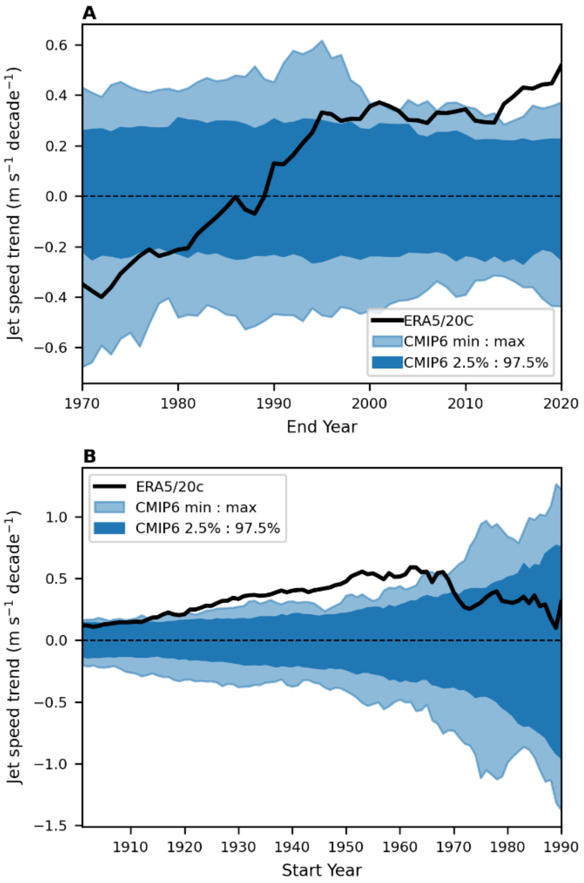

Fig. S5. Comparison of observed jet speed trends to model trend distributions. (A) The magnitude of linear trends in jet speed for all overlapping 70-year trend as a function of end year. The black line represents trends in ERA5 concatenated with ERA20C, the light blue shading represents the full range of trends from all CMIP6 model realizations, and the dark blue shading represents the 2.5% to 97.5% range. **(B)** As in (A), but for the magnitude of linear trends in jet speed ending in 2020 as a function of start year.

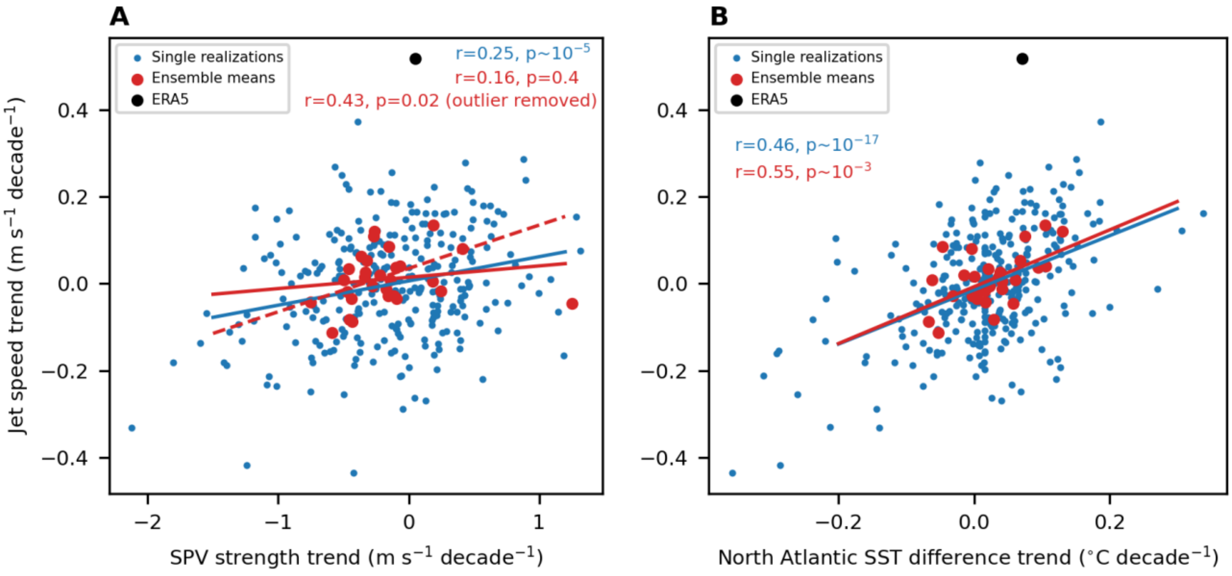

Fig. S6. The link between jet speed trend and potential drivers. (**A**) A scatter plot of jet speed trend against the stratospheric polar vortex (SPV) strength trend over the 1951-2020 period during winter. The stratospheric polar vortex is defined as the zonal mean zonal wind at 10 hPa averaged from 60°-75°N. The black dot represents ERA5, the blue dots indicate the individual ensemble members from CMIP6, and the red dots indicate the model means for models with two or more ensemble members. The blue and red lines indicate the linear fits, and the correlation (r) and p-values are indicated. The dashed red line indicates the linear fit with the one outlier model removed. (**B**) As in (A) but for jet speed trend against the meridional North Atlantic SST difference trend calculated as the difference between the average SST between 70°-40°W, 26°-40°N and 50°-30W°,45°-58°N.

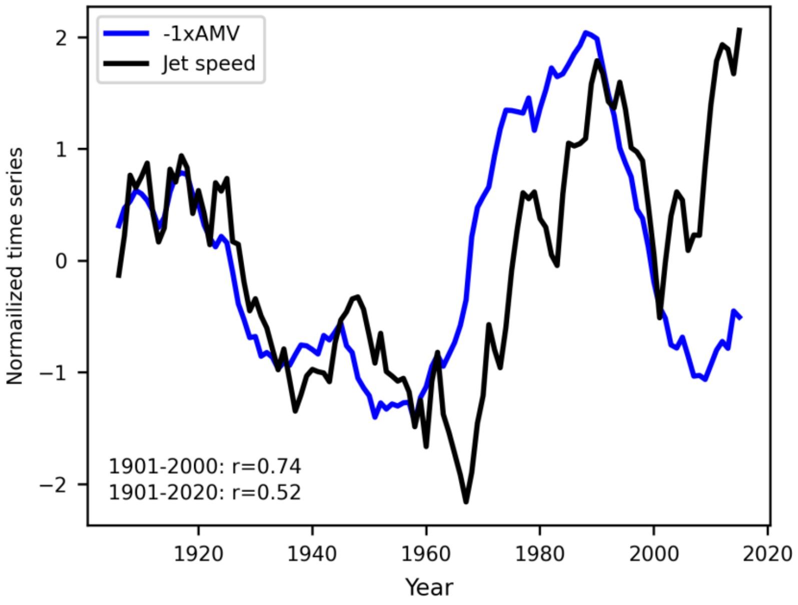

Fig. S7. The link between jet speed and AMV. Normalized time series of the winter jet speed (black) and the winter AMV index (blue). Both time series are 11-year running means and the AMV index is multiplied by -1 to make the comparison with the jet speed easier. The jet speed time series is ERA20C concatenated with ERA5. The correlations between the two time series for 1901-2000 and 1901-2020 are listed in the bottom left.

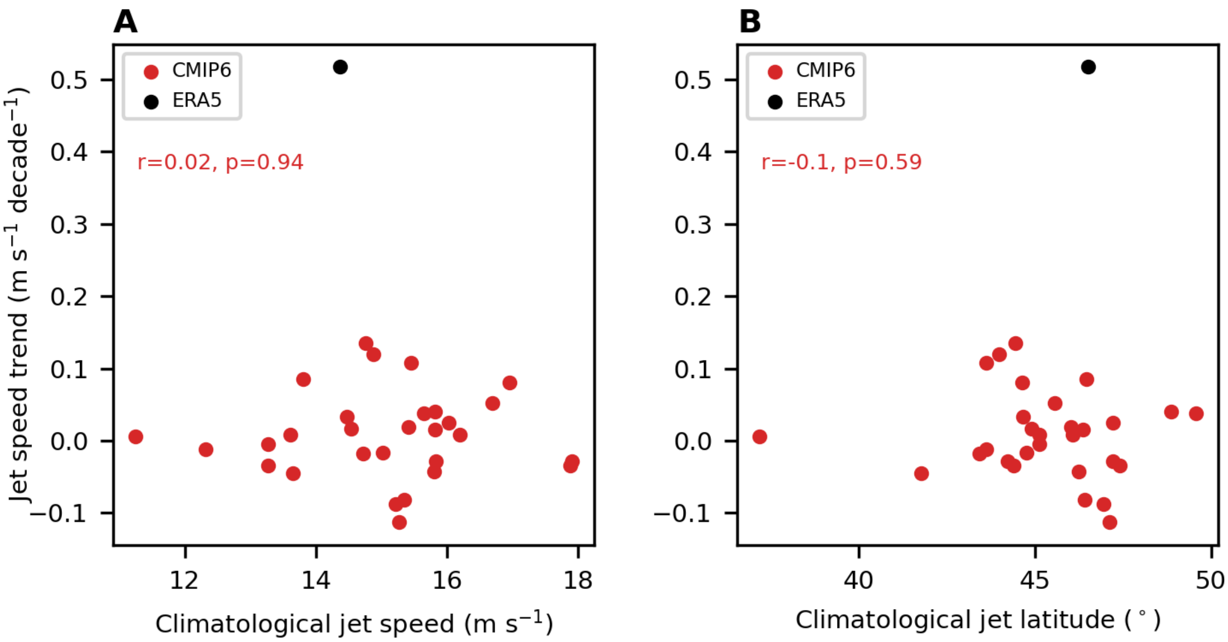

Fig. S8. Links to the biases in the climatological jet. (**A**) Scatter plot of the jet speed trend against the climatological jet speed averaged over 1951-2020 during winter. The black dot represents the ERA5 trend, and the red dots represent the models means for CMIP6 models with two or more ensemble members. The correlation (r) and p-values are indicated on the plot. (**B**) As in (A) but for climatological jet latitude.

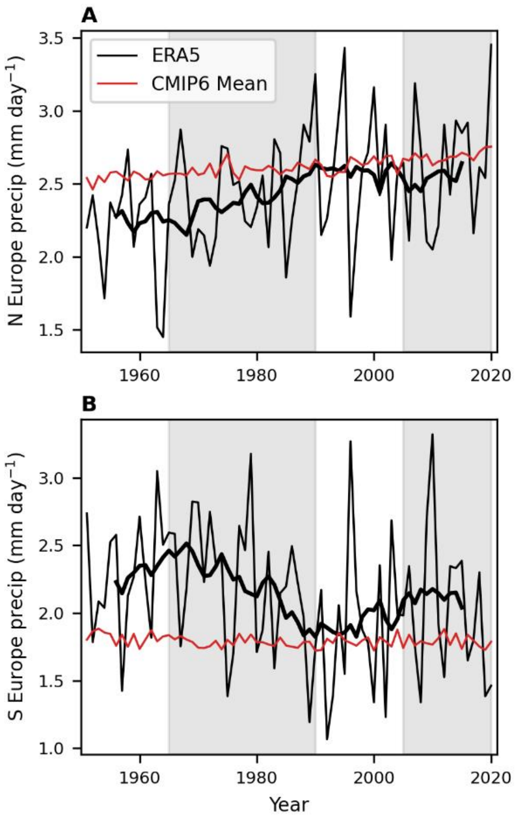

Fig. S9. Precipitation time evolution. (**A**) Time series for northern Europe precipitation (10°W-22°E, 48°N-68°N, land only). The thin black line represents ERA5, the thick black line represents an 11-year running mean of ERA5 and the thin red line represents the CMIP6 multimodel mean. The grey shading highlight the periods from 1965-1990 and from 2005-2020. (**B**) As in (A), but for southern Europe precipitation (10°W-22°E, 32°N-44°N, land-only).

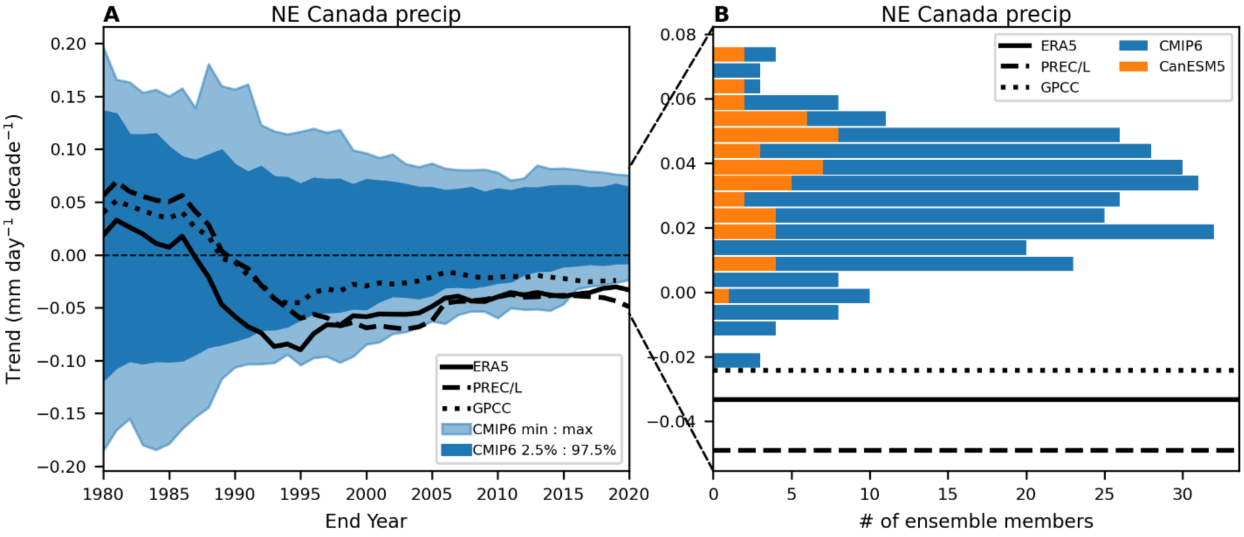

Fig. S10. Comparison of observed precipitation trends to model trend distributions.
As in Fig. 7, but for northeastern Canada precipitation.

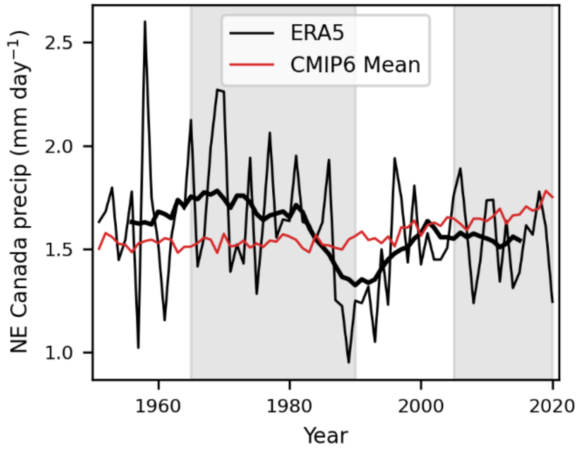

Fig. S11. Time evolution of northeastern Canada precipitation. As in Figure S9A, but for precipitation averages over northeastern Canada (75°W-60°W, 50°N-62°N, land only).

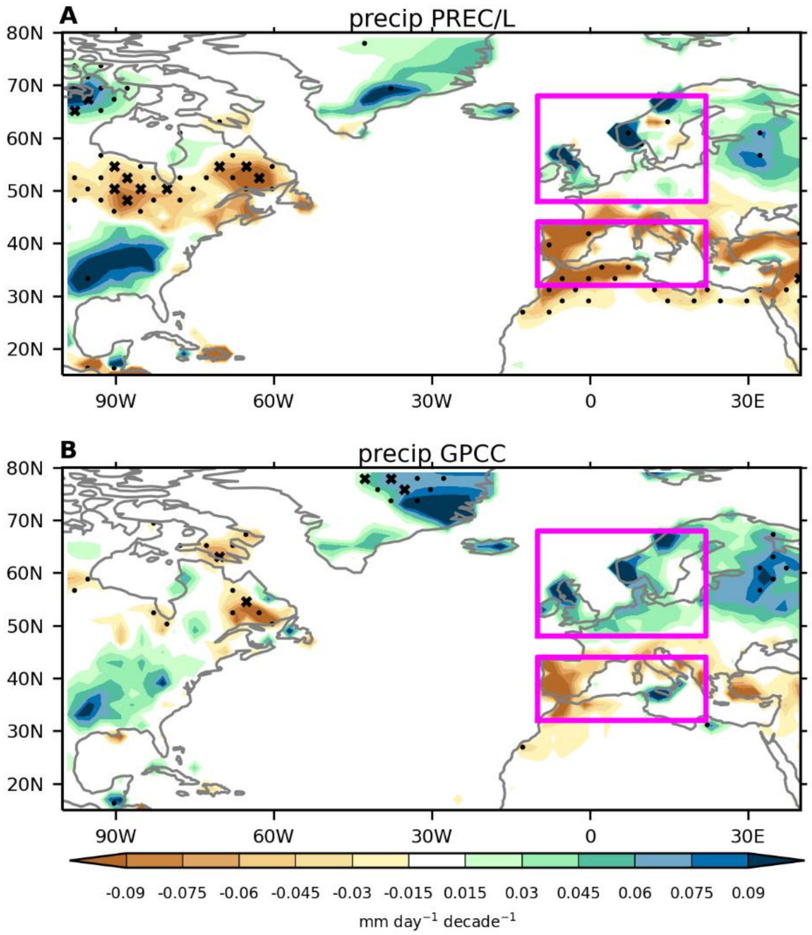

Fig. S12. Precipitation trends from observations. As in Figure 6A, but for trends in observations from (**A**) PREC/L and (**B**) GPCC.

Table S1. Summary of CMIP6 models used in the analysis.

Model name	Modelling Centre	# of ensemble members
ACCESS-CM2	CSIRO-ARCCSS	3
ACCESS-ESM1-5	CSIRO	18, 16 (SLP), 19 (precip)
AWI-CM-1-1-MR	AWI	1
BCC-CSM2-MR	BCC	1
CanESM5	CCCma	50
CanESM5-CanOE	CCCma	3
CAMS-CSM1-0	CAMS	2
CAS-ESM2-0	CAS	2, 1 (SLP)
CESM2	NCAR	3
CESM2-WACCM	NCAR	3
CIESM	THU	1
CMCC-CM2-SR5	CMCC	1
CMCC-ESM2	CMCC	1
CNRM-CM6-1	CNRM-CERFACS	10
CNRM-CM6-1-HR	CNRM-CERFACS	1
CNRM-ESM2-1	CNRM-CERFACS	9
EC-Earth3	EC-Earth-Consortium	18
EC-Earth3-CC	EC-Earth-Consortium	1
EC-Earth3-Veg	EC-Earth-Consortium	8
EC-Earth3-Veg-LR	EC-Earth-Consortium	3
FGOALS-f3-L	CAS	1
FGOALS-g3	CAS	4
FIO-ESM-2-0	FIO-QLNM	3
GFDL-CM4	NOAA-GFDL	1
GFDL-ESM4	NOAA-GFDL	3
GISS-E2-1-G	NASA-GISS	19
HadGEM3-GC31-LL	MOHC	4
IITM-ESM	CCCR-IITM	1
INM-CM4-8	INM	1
INM-CM5-0	INM	1
IPSL-CM6A-LR	IPSL	11
KACE-1-0-G	NIMS-KMA	3
KIOST-ESM	KIOST	1, 0 (precip)
MCM-UA-1-0	UA	1
MIROC6	MIROC	50
MIROC-ES2L	MIROC	30
MPI-ESM1-2-HR	MPI-M	2
MPI-ESM1-2-LR	MPI-M	10
MRI-ESM2-0	MRI	5
NESM3	NUIST	2
NorESM2-LM	NCC	3
NorESM2-MM	NCC	2
TaiESM1	AS-RCEC	1

UKESM1-0-LL	MOHC	5

Table S2: Summary of HighResMIP models used in the analysis.

Model	Modelling Centre	# of ensemble members (hist-1950)	# of ensemble members (highresSST-present)	Nominal horizontal atmospheric resolution (km)
HiRAM-SIT-LR	AS-RCEC	2		50
HiRAM-SIT-HR	AS-RCEC	1		25
FGOALS-f3-L	CAS		1	100
FGOALS-f3-H	CAS		1	25
CMCC-CM2-HR4	CMCC	1	1	100
CMCC-CM2-VHR4	CMCC	1	1	25
EC-Earth3P	EC-Earth-Consortium	3	3	100
EC-Earth3P-HR	EC-Earth-Consortium	3	3	50
ECMWF-IFS-LR	ECMWF	8	8	50
ECMWF-IFS-HR	ECMWF	6	6	25
IPSL-CM6A-LR	IPSL		1	250
IPSL-CM6A-ATM-HR	IPSL		1	50
HadGEM3-GC31-LM	MOHC		3	250
HadGEM3-GC31-HM	MOHC		3	50
HadGEM3-GC31-LL	MOHC	8		250
HadGEM3-GC31-HH	NERC	1		50
MPI-ESM1-2-HR	MPI-M	1	1	100
MPI-ESM1-2-XR	MPI-M	1	1	50
MRI-AGCM3-2-H	MRI		1	50
MRI-AGCM3-2-S	MRI		1	25
CNRM-CM6-1	CNRM-CERFACS	3	10	250
CNRM-CM6-1-HR	CNRM-CERFACS	3	10	100
